# Biodentine^TM^ versus formocresol pulpotomy technique in primary molars: a 12–month randomized controlled clinical trial

**DOI:** 10.1186/s12903-018-0702-4

**Published:** 2019-01-07

**Authors:** Omar Abd El Sadek El Meligy, Najlaa Mohamed Alamoudi, Sulaiman Mohamed Allazzam, Azza Abdel Mohsen El-Housseiny

**Affiliations:** 1 Pediatric Dentistry Department, Faculty of Dentistry, King Abdulaziz University, P.O. Box 80209, Jeddah, 21589 Kingdom of Saudi Arabia; 2 Pediatric Dentistry and Dental Public Health Department, Faculty of Dentistry, Alexandria University, Alexandria, 21521 Egypt; 3 Pediatric Dentistry Department, Faculty of Dentistry, King Abdulaziz University, P.O. Box 80209, Jeddah, 21589 Kingdom of Saudi Arabia; 4 Dental Department, Comprehensive Specialty Clinics for Security Forces, P.O. Box 1666, AlRass, 51921 Qassim Kingdom of Saudi Arabia; 5 Pediatric Dentistry Department, Faculty of Dentistry, King Abdulaziz University, P.O. Box 80209, Jeddah, 21589 Kingdom of Saudi Arabia; 6 Pediatric Dentistry and Dental Public Health Department, Faculty of Dentistry, Alexandria University, Alexandria, 21521 Egypt

**Keywords:** Biodentine^TM^, Formocresol, Primary molars, Pulpotomy, Randomized clinical trial

## Abstract

**Background:**

The objective of this clinical study was to prospectively compare the clinical and radiographic success rates of Biodentine^TM^ pulpotomies versus formocresol pulpotomies in children vital primary molars.

**Methods:**

A randomized, split–mouth study design was used with a sample of 37 healthy children aged 4– to 8–year–old. A total of 56 pairs (112 teeth) of carious primary teeth, 1 pair per child, were selected for treatment. One tooth from each pair was randomly assigned to either the Biodentine^TM^ pulpotomy group or the formocresol pulpotomy group. Children were followed–up at 3, 6 and 12 months for clinical evaluation and at 6 and 12 months for radiographic evaluation. Data were collected, tabulated and analyzed using Fisher exact and McNemar tests. The level of significance was set at P < 0.05.

**Results:**

At 12 months, the clinical success rates of both Biodentine^TM^ and formocresol groups were 100%, while the radiographic success rates for Biodentine^TM^ and formocresol groups were 100% and 98.1%, respectively. There was no statistically significant difference between both groups (P=1). The only observed radiographic failure was furcal radiolucency in the formocresol group at 12–month interval. Pulp canal obliteration was radiographically observed in 10/56 (17.9%) and 7/56 (12.5%) cases in the Biodentine^TM^ and formocresol groups, respectively.

**Conclusions:**

Both Biodentine^TM^ and formocresol pulpotomy techniques demonstrated favorable clinical and radiographic outcomes over a 12-month period without any significant difference.

**Trial registration:**

Registered on NCT03779698. Date of Registration: 18 December 2018.

## Background

Pulpotomy is defined as “the surgical removal of the coronal part of an exposed vital pulp as a means of maintaining the function and vitality of the remaining radicular pulp” [1].

Formocresol is a long–term clinically–successful medicament for use in the pulpotomy procedure, mostly due to its excellent clinical success and ease in use [2].

Several studies have been done to investigate the risk of exposure to formocresol because it has mutagenic, toxic and carcinogenic risks in humans [3–5]. Due to this, many medicaments have been prepared, examined and systemized as possible replacements for formocresol [6].

Lately, Septodont developed a new tricalcium–silicate cement (Biodentine^TM^) which could join perfect mechanical features with outstanding biocompatibility, in addition to a bioactive performance [7]. Biodentine^TM^ has been introduced and progressed (through active biosilicate technology) with the purpose of incorporating the increased biocompatibility and bioactivity of calcium silicates, producing improved features that cause it to be better than any other calcium silicate–based cements such as quick setting time, high compressive strength, and easy dealing with as well as its many uses in both endodontics and restorative dentistry without resulting in discoloration of the treated teeth [8–10]. Biodentine^TM^ drew awareness in the specialty of endodontics due to its excellent sealing ability, handling properties, biocompatibility, stability, long–term impermeability, low solubility, quick setting time, and capability to induce hard tissue regeneration. Also, it has been confirmed that Biodentine^TM^ has superior antimicrobial characteristics due to its high pH [8–10]. Furthermore, it excludes the necessity of a restorative material to fill the pulp chamber [8–11]. Therefore, Biodentine^TM^ could a good replacement to the current medicaments for regeneration of dentin–pulp complex [7].

Various in vitro and in vivo researches have confirmed the bioactivity of Biodentine^TM^, also its outstanding performance in vital pulp treatment [12–14]. Furthermore, most of the current clinical researches showed promising outcomes for its application in primary teeth leading to its use for the pulpotomy procedure in children [15–17].

Recently, most studies have concentrated on comparing formocresol to mineral trioxide aggregate (MTA) and Bioaggregate (BA) for human primary teeth pulpotomy [18–20]. Due to its excellent characteristics, in addition, its capability to control the disadvantages of both formocresol and MTA, Biodentine^TM^ could be an excellent replacement to the current materials for pulp therapy. It has the ability to create great participation in preserving the vitality of the pulp in children wisely chosen for pulpotomy procedure [7].

The literature concerning its clinical and radiographical success in primary teeth pulpotomy is few. Thus, future clinical research is required to utilize Biodentine^TM^ as a substitute to formocresol in primary teeth pulpotomy. The objective of the current research was to compare prospectively the clinical and radiographic success rates of Biodentine^TM^ pulpotomies versus formocresol pulpotomies in children vital primary teeth.

## Methods

This study was written according to the Consolidated Standards of Reporting Trials (CONSORT) statement [21].

### Study design

A double–blinded, split–mouth, randomized, controlled clinical study was done.

### Patients

This study was carried out on healthy children aged 4– to 8–year–old. The children were selected from the Pediatric Dentistry Clinics, Faculty of Dentistry, King Abdulaziz University (KAU), Jeddah. Each patient had at least 2 matched bilateral carious primary molars requiring pulpotomy. Each parent signed an informed consent for the child’s participation in the study. No children were excluded based on gender, race, social or economic status.

Teeth were selected based upon the following clinical and radiographic criteria: Clinically, the study included teeth with restorable crowns, teeth with pathologic carious or mechanical exposure of vital pulps and teeth with no clinical symptoms or evidence of pulp degeneration, such as spontaneous or nocturnal pain, pain on percussion, history of swelling, or sinus tracts and teeth with no tenderness to percussion, physiologic or pathologic mobility. Radiographically, the recruited teeth should have a normal radiographic appearance with healthy supporting tissues, no signs of internal resorption, or pathologic external root resorption and no periapical or inter–radicular pathosis, with at least two–thirds of the root remaining (not more than one–third of the root is physiologically resorbed). Teeth were unselected if any of the previously–stated criteria were not met.

Preoperative periapical radiographs of the molars considered for treatment were taken using the XCP extension cone paralleling technique.

### Sample size and power determination

Sample size calculation for binary outcome equivalence trials was calculated using sample size calculators of a sealed envelope, randomization and online databases for clinical trials at https://www.sealedenvelope.com/power/binary-equivalence/

Thus, if there is truly no difference between the standard and experimental materials, then 102 teeth (51 for each study material) are required to be 95% sure that the limits of a two–sided 90% confidence interval will exclude a difference between the standard and experimental group of more than 10%. It is usually prudent to plan to include more than the minimum number of teeth in a study to compensate for loss during follow–up or other causes of attrition. The percentage of teeth that could be lost to follow up at all stages was set at 10% thereby forcing an increase of 5 pairs to the calculated sample size. Thus, the final sample size for this study was calculated to be 112 teeth.

### Randomization

Since the teeth indicated for pulpotomy must be treated as soon as possible, the patients were included at the time of diagnosis (identification) and randomization for the materials on the sides was done. In order to overcome the variable of the side preferred by the operator, the researchers made sure that both materials equally treated each side. This was performed by carrying out the block randomization technique with closed envelopes. Before recruitment of the patients, 56 sealed envelopes containing the result of randomization were prepared, sealed, and blindly mixed in a box. An envelope was for each block of two contralateral teeth (one pair). After that, the envelopes were numerated blindly from 1 to 56. The envelopes were assigned to the 56 pairs according to the beginning of the treatment (envelop number 1 was assigned to the earliest pair ready for treatment and so on). Each envelope was unsealed after the signature of the informed consent and immediately before the implementation of the first procedure on the right tooth.

One hundred and twelve molars were randomly divided into two treatment groups. Group I comprised 56 molars treated with Biodentine^TM^ (experimental). Group II comprised 56 molars treated with formocresol (control). Each patient received 2 treatments, Biodentine^TM^ on one side of the oral cavity and formocresol on the other side.

### Procedures

An operator performed the pulpotomy procedures. After application of topical anesthesia (Beutlich LP Pharmaceuticals, USA), local anesthesia was administrated using 27–gauge short needles and syringes loaded with carpules, each one contained 1.8 ml of Lidocaine 2% with epinephrine concentration of 1:100000 (Octocaine® 100, Novocol Healthcare Inc. Cambridge, Ontario, Canada). Complete isolation was performed using a rubber dam and saliva ejector. Removal of caries and deroofing of the pulp chamber were performed using a no. 330 high–speed carbide bur with copious water spray. A sharp sterile spoon excavator or a slow–speed round carbide bur (no. 6 or no. 8) was used for coronal pulp amputation. Then the pulp chamber was washed with normal saline and bleeding was controlled by placing a cotton pellet moistened with water in the pulp chamber for 5 minutes.

In the experimental group (group I), Biodentine^TM^ (Septodont Ltd., Saint Maur des Faussés, France) was used following the manufacturer’s recommendations. The whole pulp chamber was entirely filled with Biodentine^TM^ until the occlusal surface. In control (group II), a sterile cotton pellet moistened with 1:5 concentration formocresol (Buckley’s Formocresol, Sultan Healthcare, Englewood, NJ, USA) then blotted to remove excess was placed for 5 minutes on the pulp stumps and then the pulps were covered with zinc oxide–eugenol (IRM; Dentsply, Milford, DE) dressing. In both groups, all teeth were finally restored using a stainless steel crown (SSC) (3M/ESPE, St. Paul, Minn., USA).

Follow–up was done for all children clinically at 3, 6 and 12 months and radiographically at 6 and 12 months. Two full–time pediatric dentistry faculty members (other than the operator) from KAU blindly evaluated all the teeth clinically and radiographically.

### Outcome assessment criteria

The pulpotomy procedure was decided a clinical success if the tooth fulfilled the following criteria [1, 18, 19, 22]: (1) No pain, (2) No swelling, (3) No tenderness to percussion, (4) No abscess or fistula, and (5) No abnormal tooth mobility. The pulpotomized tooth was judged to be radiographically successful if it demonstrated the following criteria [1,18,19,22, 1) Normal periodontal ligament space (2) No periapical and furcation pathosis, and (3) No internal resorption. If pulp canal obliteration (PCO) happened, it was recorded but not considered as a treatment failure [23].

### Statistical analysis

Data were statistically analyzed using the Statistical Package for Social Sciences (SPSS) version 20.0 (SPSS Inc., Chicago, IL). Inter– and intra–examiner agreement was performed using the Kappa statistic. Fisher exact test was used to assess differences in success rates between both groups at 3, 6 or 12 months. McNemar’s test was used to compare these rates in each group between pairs of follow up periods. The level of significance was set at P < 0.05.

### Ethical Considerations

Ethical approval was obtained from the Research Ethics Committee, Faculty of Dentistry, KAU, Jeddah, Kingdom of Saudi Arabia (Approval no. 029–14).

## Results

### Demographic characteristics

**Fig. 1:**
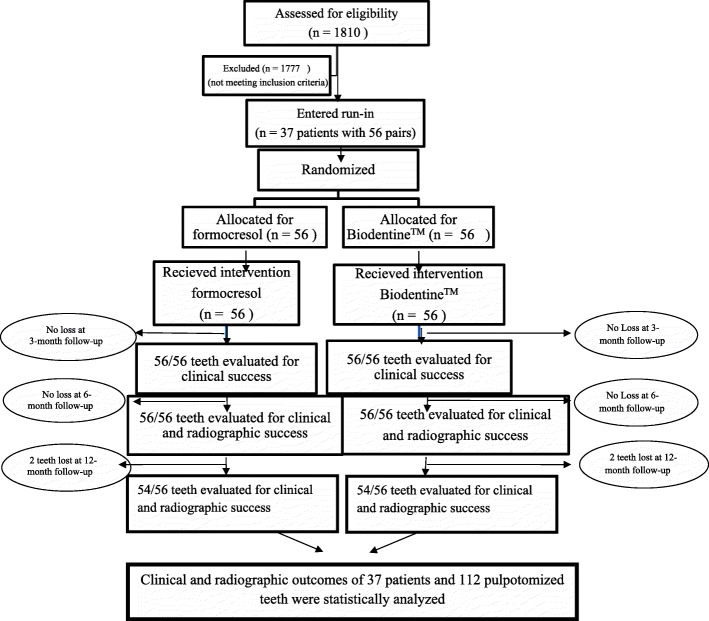
A CONSORT diagram showing the study protocol up to the 12–month follow–up

All 112 (100%) primary molars were available for 3– and 6–month clinical evaluation and 6–month radiographic evaluation. One patient with 4 (3.6%) pulpotomized teeth (2 pairs) was unavailable for the 12–month evaluation. Hence, 108 teeth were clinically and radiographically evaluated at the end of 12 months. A CONSORT diagram showing the flow of patients and pulpotomized teeth up to 12–month follow–up is presented in Fig. 1 [21].

The children age at the time of treatment ranged between 4–8 years (mean age, 6 ± 0.75 years). As the split–mouth method was carried out, no difference between both groups (Biodentine^TM^ and formocresol) concerning the patient’s age at the treatment time, gender and in the kind of molar treated was found. Generally, lower molars (79%) were more frequently recruited than upper ones (21%). The majority of the treated teeth were the lower second molar (44.6%), then the lower first molar (34%), upper second molar (16%), and upper first molar (5.4%). Table 1 shows the sample distribution of the evaluated teeth.

**Table 1:** Baseline data of the study sample

	Patients n (%)	Pairs n (%)	Teeth n (%)
Total	37	56	112
Males	17 (45.9)	24 (42.9)	48 (42.9)
Females	20 (54.1)	32 (57.1)	64 (57.1)
4 years	5 (13.5)	8 (14.3)	16 (14.3)
5 years	11 (29.7)	17 (30.4)	34 (30.4)
6 years	13 (35.1)	18 (32.1)	36 (32.1)
7 years	4 (10.8)	6 (10.7)	12 (10.7)
8 years	4 (10.8)	7 (12.5)	14 (12.5)
Maxillary arch	-	-	24 (21.4)
Mandibular arch	-	-	88 (78.6)
Primary first molar	-	-	44 (39.3)
Primary second molar	-	-	68 (60.7)

Clinical calibration results by the 2 examiners were considered excellent (k=0.98). Regarding radiographical calibration, inter–examiner (k=0.97) and intra–examiner agreement (k=0.98 and 0.97 for the 2 examiners) was also considered excellent.

### Clinical success

After 3–, 6–, and 12– months, Biodentine^TM^ and formocresol groups showed 100% clinical success rates (Table 2). No statistically significant differences were recorded between any of the groups at 3, 6, and 12 months. All teeth were free from abscess formation, mobility or a draining sinus at all the intervals.

**Table 2:** Clinical and radiographic success rates of Biodentine^TM^ and formocresol groups at 3–, 6–, and 12–month

Assessment		3 months n (%)	6 months n (%)	12 months n (%)	P of McNemar’s test (3/6 to 12 months)
Clinical	Biodentine^TM^	56/ 56 (100)	56/ 56 (100)	54/ 54 (100)	-
	Formocresol	56/ 56 (100)	56/ 56 (100)	54/ 54 (100)	-
P of Fisher exact test		-	-	-	
Radiographic	Biodentine^TM^	-	56/ 56 (100)	54/ 54 (100)	-
	Formocresol	-	56/ 56 (100)	53/ 54 (98.1)	1.00
P of Fisher exact test		-	-	1.00	

Clinically, at all follow–up periods and radiographically at 6 and 12 months in both groups, all cases were successful, and no test was computed

### Radiographic success

**Fig. 2:**
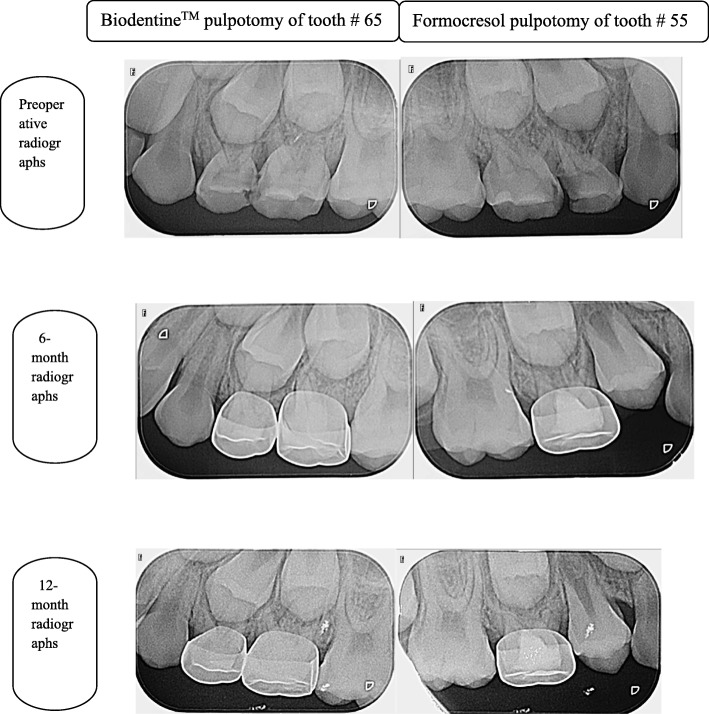
Periapical radiographs showing successful Biodentine^TM^ pulpotomy of tooth 65 and formocresol pulpotomy of tooth 55 (maxillary second primary molars)

The Biodentine^TM^ group had a radiographic success rate of 100% at 6 and 12–month follow–up periods, while the formocresol group had a radiographic success rate of 100% at 6 months, followed by 98.1% at 12 months (Table 2). No statistically significant differences were noted between any of the groups at 6 and 12 months. No pathological signs were observed radiographically in any of the 2 groups except for one molar in the formocresol group that showed furcation radiolucency at 12–month interval. PCO was observed in 10/56 cases (17.8%) in the Biodentine^TM^ group, and in 7/56 cases (12.5%) in the formocresol group. Figure 2 shows radiographs of one successfully treated tooth for each of the 2 study groups.

## Discussion

The importance of pulp vitality preservation can never be overstated. Selection of the method used in vital pulp therapy relies on the degree of inflammation of the pulp. Pulpotomy is the favored method only if the coronal part of an exposed vital pulp is inflamed because of infiltration of microorganisms after carious or mechanical reasons, and the remaining radicular pulp is healthy [24]. Despite the success rate of indirect pulp treatment for managing extensive carious lesions approximating the pulp in primary teeth, practitioners still hesitate to practice this technique because of lack of studies and strong evidence on this topic [25].

This current randomized, split–mouth clinical study is considered as one of the few prospective clinical trials that used Biodentine^TM^ in pulpotomy of primary molars in children. It was conducted to evaluate the success rate of Biodentine^TM^ pulpotomy technique in human primary molars, clinically and radiographically and compare it with formocresol pulpotomy technique.

The control pulpotomy technique used in this study “formocresol” was selected because it is a long–term clinically–successful medicament, even though its adverse reactions remain a concern [2, 26]. Also, formocresol is used for pulpotomies of primary teeth in Pediatric Dental Clinics, Faculty of Dentistry, KAU, Jeddah. Although MTA biological approach and its performance is comparable or similar to formocresol, it has some disadvantages such as prolonged setting time, difficult handling characteristics and high material cost [27–29]. When comparing formocresol to ferric sulfate, a recent systematic review indicates that the total success rate at 2 years for formocresol was 87.1% and ferric sulfate was 84.8%, with the meta–analysis favoring neither agent’s success [30]. Biodentine^TM^ was investigated in the current study because it was recently marketed and showed success in several clinical applications such as endodontic and restorative dentistry [8–10]. Because of its regenerative properties, it was suggested to be used as a suitable replacement to formocresol in pulpotomy of primary teeth [31].

Since Biodentine^TM^ showed success in many endodontic procedures and used as a permanent dentine substitute, the pulp chamber was filled up to the occlusal level with Biodentine^TM^ [32]. This may give long–standing advantages on improving the evaluation and performance of primary molars pulpotomy [9].

Preoperative periapical radiographs were used as a baseline for comparisons in different follow–ups and to assess the periapical area. It is advisable to use radiographs that clearly reveal the periradicular areas to monitor prognosis of primary molars pulpotomy, because the failure of pulpotomy in these teeth may be demonstrated in the furcation or periapical areas [33].

Results of the current research revealed high clinical and radiographic success rates at all observation periods for the 2 medicaments used; this might be due to correct protocol, complete isolation and tooth selection, good aseptic conditions, and appropriate manipulation for the material. These results are in accordance with that of a 6–month follow–up previous randomized controlled clinical study, which compared Biodentine^TM^ to formocresol in pulpotomy of primary teeth [31]. No significant difference was found between the two techniques, in that trial, with a success rate of 100% for Biodentine^TM^ and 94% for formocresol, suggesting that, Biodentine^TM^ is an alternative to formocresol in primary teeth pulpotomy.

Results of the current study also agree with the success rates of formocresol reported at one year in previous researches [1, 18, 19, 22]. This increased success rate of formocresol in the current research is attributed to the precise criteria used for teeth selection and to the antiseptic (germicidal) and fixative properties of formocresol. Furthermore, this high success rate of formocresol is not in agreement with the results of other studies [34, 35]; because those studies had smaller sample sizes.

In our study, the success rate of Biodentine^TM^ (100%) was comparable to the success rate reported in previous studies [14, 36]. They were also similar to a recent study, which found clinical and radiographic success rates of 95.24% and 94.4% respectively for this novel material, but with a longer follow–up period and a smaller sample size [37]. This may be attributed to the excellent sealing and regeneration abilities, the higher biocompatibility and alkalinity of Biodentine^TM^ that may lead to this increased success rate. In addition, these outcomes are also confirmed and described by a histological observation in pigs’ primary teeth that when placed against pulp stumps following pulpotomy, Biodentine^TM^ boosted beneficial calcification [12].

At 6 and 12 months in both materials, PCO was the only radiographic finding detected in 15.2% (17/112) of all teeth. PCO was observed in 10 (17.8%) molars in the Biodentine^TM^ group and just 7 (12.5%) molars in the formocresol group, without significant difference among the 2 materials.

PCO is a usual radiographic observation in pulpotomy procedures using formocresol, ferric sulfate, or Biodentine^TM^ [1, 19, 34]. PCO was detected in a wide range (0–52%) of teeth treated by formocresol pulpotomy [1, 18, 19, 22, 34, 38]. In the current study, formocresol technique showed PCO in only 12.5% of molars, which agrees with the percentage found in published studies. The present result of 17.9% for PCO in the Biodentine^TM^ technique was in the range observed as in MTA [1, 19, 22, 37]. PCO results from vigorous odontoblastic activity and indicate pulpal vitality.

In addition to the high success rate of Biodentine^TM^ observed in the current study, it has a clinical advantage over formocresol that is the simultaneous action of Biodentine^TM^ as both a dressing and restorative material. While, formocresol requires a restorative material in the pulp chamber since it serves only as a pulpotomy medicament [32].

The current study has some limitations, the limited available time for follow up, strict inclusion criteria for selecting participants. Unfortunately, operator blinding was not possible as both materials were of different types, which required different manipulations, thus the operators’ cognitive bias could not be eliminated during the procedures but not in the follow–up evaluations.

The current study has some advantages as it is one of the few recent randomized clinical trials using Biodentine^TM^ for primary molars pulpotomy. The sample size was adequate. There were no significant differences in clinical and radiographic success rates between Biodentine^TM^ and formocresol. It is suggested that Biodentine^TM^ is a promising material with a high success rate without any adverse effects in all pulpotomized molars. Our study shows that Biodentine^TM^ has the power to be an alternative for formocresol in primary teeth pulpotomy. Although the number of participants was sufficient, it is premature to draw a final conclusion, because of the short follow–up period. This clinical trial might provide a base for more research with more participants and longer follow–up periods.

The improved properties of Biodentine^TM^, as well as its simple manipulation, may motivate dental practitioners to utilize this contemporary material as a practical choice in primary molars pulpotomy.

## Conclusions

Both Biodentine^TM^ and formocresol pulpotomy techniques demonstrated favorable clinical and radiographic outcomes in human primary molar teeth over a 12-month period without any significant difference.

## Abbreviations

MTA: Mineral trioxide aggregate
BA: Bioaggregate
CONSORT: Consolidated Standards of Reporting Trials
KAU: King Abdulaziz University
SSC: Stainless steel crown
PDL: Periodontal ligament
PCO: Pulp canal obliteration
SPSS: Statistical Package for Social Sciences

## References

[1] Erdem A, Guven Y, Balli B, Ilhan B, Sepet E, Ulukapi I, Aktoren O (2011). Success rates of mineral trioxide aggregate, ferric sulfate and formocresol pulpotomies: a 24–month study. Pediatr Dent..

[2] American Academy of Pediatric Dentistry (2018). Pulp therapy for primary and immature permanent teeth. Pediatr Dent..

[3] Srinivasan V, Patchett CL, Waterhouse PJ (2006). Is there life after Buckley's formocresol? Part I – a narrative review of alternative interventions and materials. Int J Paediatr Dent..

[4] Lewis B (2010). The obsolescence of formocresol. J Calif Dent Assoc..

[5] Athanassiadis B, George GA, Abbott PV, Wash LJ (2015). A review of the effects of formaldehyde release from endodontic materials. Int Endod J..

[6] American Academy of Pediatric Dentistry (2017). Pulp therapy for primary and immature permanent teeth. Pediatr Dent..

[7] Zanini M, Sautier J, Berdal A, Simon S (2012). Biodentine^TM^ induces immortalized murine pulp cell differentiation into odontoblast–like cells and stimulates biomineralization. J Endod..

[8] Bani M, Elif S, Odabas ME (2015). Efficacy of Biodentine^TM^ as an apical plug in nonvital permanent teeth with open apices: an in vitro study. BioMed Research International..

[9] Yoldaş SE, Bani M, Atabek D, Bodur H (2016). Comparison of the potential discoloration effect of Bioaggregate, Biodentine^TM^, and white mineral trioxide aggregate on bovine teeth: in vitro research. J Endod..

[10] Bhavya B, Sadique M, Simon EP, Ravi SV, Lal S (2017). Spectrophotometric analysis of coronal discoloration induced by white mineral trioxide aggregate and Biodentine^TM^: an in vitro study. J Conserv Dent..

[11] Shamkhalov GS, Ivanova EV, Dmitrieva NA, Akhmedova ZR (2013). Comparative study of antimicrobial activity of "Biodentine^TM^ " and "Rootdent" cements and "Futurabond NR" adhesive. J Stomato..

[12] Han L, Okiji T (2013). Bioactivity evaluation of three calcium silicate–based endodontic materials. Int Endod J..

[13] Nowicka A, Lipski M, Parafiniuk M, Sporniak–Tutak K, Lichota D, Kosierkiewicz A, Kaczmarek W, Buczkowska–Radlińska J (2013). Response of human dental pulp capped with Biodentine^TM^ and mineral trioxide aggregate. J Endod..

[14] Bani M, Aktaş N, Çınar Ç, Odabaş ME (2017). The clinical and radiographic success of primary molar pulpotomy using Biodentine™ and mineral trioxide aggregate: a 24-month randomized clinical trial. Pediatr Dent..

[15] Niranjani K, Prasad MG, Vasa AA, Divya G, Thakur MS, Saujanya K (2015). Clinical evaluation of success of primary teeth pulpotomy using mineral trioxide aggregate(®), laser and Biodentine(TM) – an in vivo study. J Clin Diagn Res..

[16] Cuadros-Fernández C, Lorente Rodríguez AI, Sáez-Martínez S, García-Binimelis J, About I, Mercadé M (2016). Short-term treatment outcome of pulpotomies in primary molars using mineral trioxide aggregate and Biodentine^TM^: a randomized clinical trial. Clin Oral Investig..

[17] Juneja P, Kulkarni S (2017). Clinical and radiographic comparison of Biodentine^TM^ , mineral trioxide aggregate and formocresol as pulpotomy agents in primary molars. Eur Arch Paediatr Dent..

[18] Agamy H, Bakry N, Mounir M, Avery D (2004). Comparison of mineral trioxide aggregate and formocresol as pulp–capping agents in pulpotomized primary teeth. Pediatr Dent..

[19] Farsi N, Alamoudi N, Balto K, Mushayt A (2005). Success of mineral trioxide aggregate in pulpotomized primary molars. J Clin Pediatr Dent..

[20] Sushynski J, Zealand C, Botero TM, Boynton JR, Majewski RF, Shelburne CE, Hu JC (2012). Comparison of gray mineral trioxide aggregate and diluted formocresol in pulpotomized primary molars: a 6– to 24–month observation. Pediatr Dent..

[21] Schulz KF, Altman DG, Moher D, CONSORT Group (2011). CONSORT 2010 statement: updated guidelines for reporting parallel group randomized trials. Int J Surg.

[22] Sonmez D, Sari S, Cetinbas T (2008). A comparison of four pulpotomy techniques in primary molars: a long-term follow–up. J Endod..

[23] Fuks A, Bimstein E, Klein H, Guelmann M (1990). Assessment of a 2% buffered glutaraldehyde solution in pulpotomized primary teeth of school children. J Dent Child..

[24] Kusum B, Rakesh K, Richa K (2015). Clinical and radiographical evaluation of mineral trioxide aggregate, Biodentine^TM^ and propolis as pulpotomy medicaments in primary teeth. Restor Dent Endod..

[25] Smaïlfaugeron V, Porot A, Mullerbolla M, Courson F (2016). Indirect pulp capping versus pulpotomy for treating deep carious lesions approaching the pulp in primary teeth:a systematic review. Eur J Paediatr Dent..

[26] Chandrashekhar S, Shashidhar J (2014). Formocresol, still a controversial material for pulpotomy: a critical literature review. J Res Dent..

[27] Glickman GN, Koch KA (2000). 21st–century endodontics. J Am Dent Assoc..

[28] Bakland LK (2000). Management of traumatically injured pulps in immature teeth using MTA. J Calif Dent Assoc.

[29] Schwendicke F, Brouwer F, Stolpe M (2015). Calcium hydroxide versus mineral trioxide aggregate for direct pulp capping: a cost–effectiveness analysis. J Endod..

[30] Coll JA, Seale NS, Vargas K, Marghalani AA, Al Shamali S, Graham L (2017). Primary tooth vital pulp therapy: a systematic review and meta–analysis. Pediatr Dent..

[31] Rubanenko M, Moskovitz M, Petel R, Fuks A (2014). Effectiveness of Biodentine^TM^ versus formocresol as dressing agents in pulpotomized primary molars: preliminary results.

[32] Priyalakshmi S, Ranjan M (2014). Review on Biodentine^TM^–a bioactive dentin substitute. J Dent Med Sci..

[33] Reddy MS (1992). Radiographic methods in the evaluation of periodontal therapy. J Periodontol..

[34] Holan G, Eidelman E, Fuks A (2005). Long–term evaluation of pulpotomy in primary molars using mineral trioxide aggregate or formocresol. Pediatr Dent..

[35] Jabbarifar S, Khademi A, Ghasemi D (2004). Success rate of formocresol versus mineral trioxide aggregate in human primary molar tooth. J Res Med Sci..

[36] Awawdeh L, Al-Qudah A, Hamouri H, Chakra RJ (2018). Outcomes of vital pulp therapy using mineral trioxide aggregate or Biodentine^TM^: a prospective randomized clinical trial. J Endod..

[37] Rajasekharan S, Martens LC, Vandenbulcke J, Jacquet W, Bottenberg P, Cauwels RG (2017). Efficacy of three different pulpotomy agents in primary molars: a randomized control trial. Int Endod J..

[38] Zealand C, Briskie D, Botero T, Boynton J, Hu J (2010). Comparing gray mineral trioxide aggregate and diluted formocresol in pulpotomized human primary molars. Pediatr Dent..

